# PARP1-MGMT complex underpins pathway crosstalk in O^6^-methylguanine repair

**DOI:** 10.1186/s13045-022-01367-4

**Published:** 2022-10-14

**Authors:** Jodie D. Cropper, Dauren S. Alimbetov, Kevin T. G. Brown, Rostislav I. Likhotvorik, Andrew J. Robles, James T. Guerra, Boxue He, Yidong Chen, Youngho Kwon, Raushan T. Kurmasheva

**Affiliations:** 1 Greehey Children’s Cancer Research Institute, University of Texas Health Science Center at San Antonio, San Antonio, TX 78229 USA; 2 Department of Population Health Sciences, University of Texas Health Science Center at San Antonio, San Antonio, TX 78229 USA; 3 Department of Biochemistry and Structural Biology, University of Texas Health Science Center at San Antonio, San Antonio, TX 78229 USA; 4 Department of Molecular Medicine, University of Texas Health Science Center at San Antonio, San Antonio, TX 78229 USA

**Keywords:** DNA damage and repair, Protein interaction, PARP1, MGMT, O^6^-Methylguanine, Cancer therapy, Ewing sarcoma

## Abstract

DNA lesions induced by alkylating agents are repaired by two canonical mechanisms, base excision repair dependent on poly(ADP) ribose polymerase 1 (PARP1) and the other mediated by O^6^-methylguanine (O^6^meG)-DNA methyltransferase (MGMT) in a single-step catalysis of alkyl-group removal. O^6^meG is the most cytotoxic and mutagenic lesion among the methyl adducts induced by alkylating agents. Although it can accomplish the dealkylation reaction all by itself as a single protein without associating with other repair proteins, evidence is accumulating that MGMT can form complexes with repair proteins and is highly regulated by a variety of post-translational modifications, such as phosphorylation, ubiquitination, and others. Here, we show that PARP1 and MGMT proteins interact directly in a non-catalytic manner, that MGMT is subject to PARylation by PARP1 after DNA damage, and that the O^6^meG repair is enhanced upon MGMT PARylation. We provide the first evidence for the direct DNA-independent PARP1-MGMT interaction. Further, PARP1 and MGMT proteins also interact via PARylation of MGMT leading to formation of a novel DNA damage inducible PARP1-MGMT protein complex. This catalytic interaction activates O^6^meG repair underpinning the functional crosstalk between base excision and MGMT-mediated DNA repair mechanisms. Furthermore, clinically relevant ‘chronic’ temozolomide exposure induced PARylation of MGMT and increased binding of PARP1 and MGMT to chromatin in cells. Thus, we provide the first mechanistic description of physical interaction between PARP1 and MGMT and their functional cooperation through PARylation for activation of O^6^meG repair. Hence, the PARP1-MGMT protein complex could be targeted for the development of advanced and more effective cancer therapeutics, particularly for cancers sensitive to PARP1 and MGMT inhibition.

**Supplementary Information:**

The online version contains supplementary material available at 10.1186/s13045-022-01367-4.

**To the editor,**

Therapeutic synergy induced by PARP1 inhibition combined with DNA alkylation has been reported by several groups [1, 2]. However, we recently demonstrated that despite the antitumor activity in Ewing sarcoma xenografts, half of the tested models were resistant to the combination of talazoparib (PARP1 inhibitor) and temozolomide (standard-of-care DNA alkylating agent) [3]. Exome sequencing analysis revealed no genetic alterations associated with this response. To guide the rational development of more effective cancer therapeutics targeting PARP1 and MGMT mechanisms responsible for repair of alkylation DNA damage, one approach is to understand how cells process DNA lesions [3–5]. It is generally thought that PARP1-mediated base excision repair (BER) and MGMT represent two distinct mechanisms for removing DNA damage induced by temozolomide [6]. In this study, we demonstrate that these mechanisms are physically coordinated, indicative of functional pathway crosstalk.

To determine cellular response to pharmacologic and genetic ablation of PARP1 and MGMT in the presence of induced DNA damage (temozolomide), cell viability assays were done on Ewing sarcoma cell lines (Fig. 1a–h). We observed that PARP1 and MGMT inhibition (by talazoparib and O^6^-benzylguanine) (Fig. 1a, b; Additional File 1: Fig. S1a, c, d) or *MGMT* gene knockdown (by RNAi) (Fig. 1c, e, f; Additional File 1: Fig. S2a, b) induced cell sensitization to temozolomide (up to 20-fold inhibition). We surmise that PARP1 and MGMT may act in a linear pathway of DNA repair in Ewing sarcoma cells and observe no correlation between PARP1-DNA trapping potency and cell sensitization to temozolomide by the two other PARP1 inhibitors, veliparib and olaparib (Additional File 1: Fig. S1b).

**Fig. 1:**
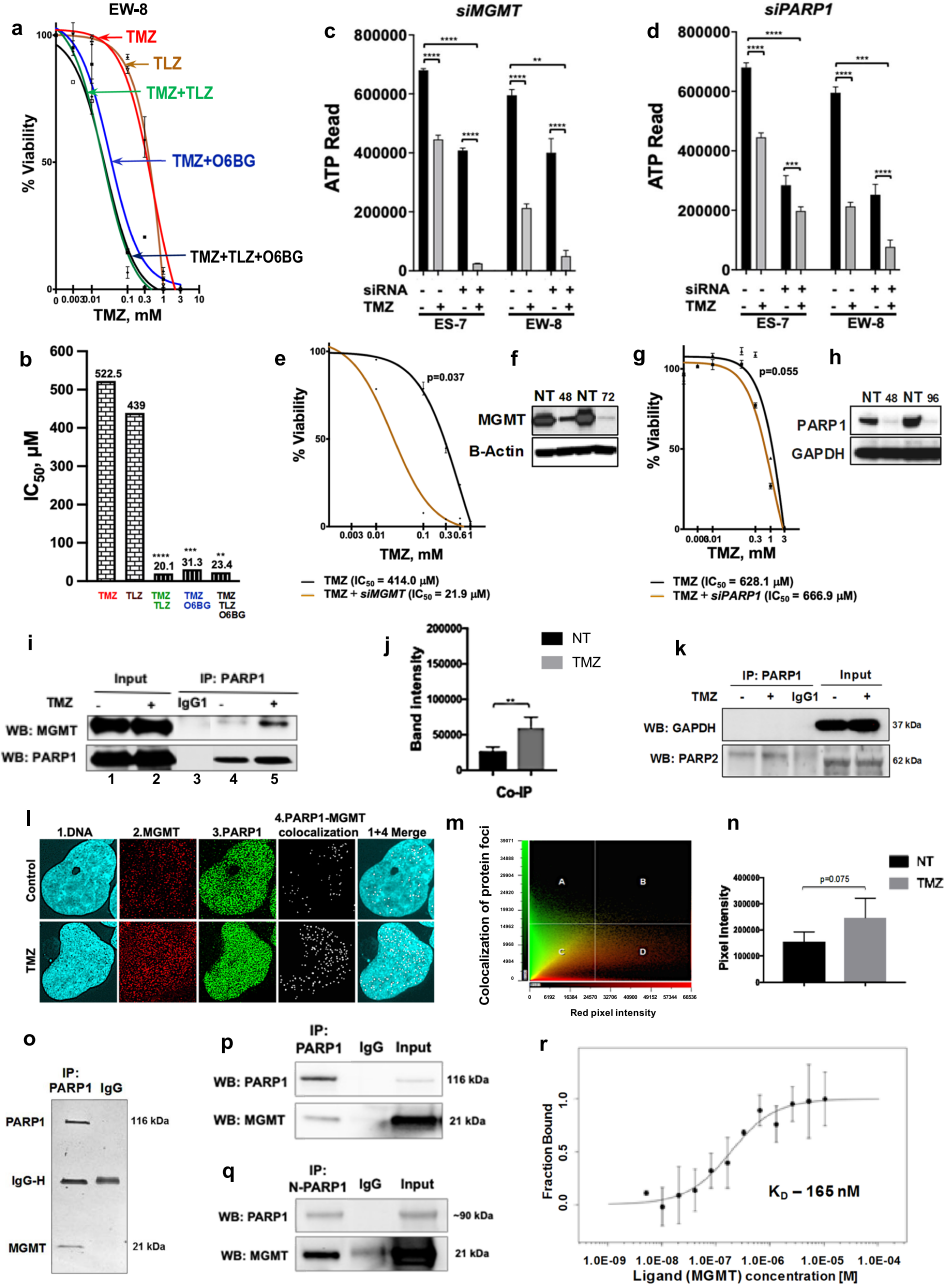
Pharmacological and genetic inhibition of PARP1 and MGMT potentiates temozolomide cytotoxicity in a linear fashion and is associated with PARP1-MGMT interaction. **a** TMZ-treated (0–3 mM) Ewing sarcoma cell lines exposed to TLZ (IC_10_) and O^6^BG (5 μM) for 96 h (Alamar Blue assay). EW-8 cell line is shown as a model example, additional results for ES-4, ES-6, and ES-7 cell lines are available in Additional File 1: Fig. S1a**.** TLZ, talazoparib. TMZ, temozolomide. O^6^BG, O^6^-benzylguanine. **b** Potentiation to TMZ: IC_50_ values for EW-8 cell line as in **a**. EW-8 cells are intrinsically resistant to TLZ [8]. P-values are calculated for TMZ *vs* TMZ + TLZ, TMZ + O^6^BG, TMZ + TLZ + O^6^BG by ANOVA3 followed by Tukey’s test for multiple comparisons: ****p ≤ 0.0001; ***p ≤ 0.001; **p ≤ 0.01; *p ≤ 0.05. Legend colors are coordinated with colors in **a**. **c** *MGMT* and **d** PARP1 gene knockdown-induced potentiation to TMZ (IC_50_, 48 h) in ES-7 and EW-8 cells (RNAi high-throughput screen). Readout is ATPlite cell viability assay. Each bar represents mean IC_50_; error bars are calculated for 3 siRNAs run in triplicate. P-values calculated by t-test, non-paired, un-equal variance, 2-sided: ****p ≤ 0.0001; ***p ≤ 0.001. *PARP1* gene knockdown was not as effective as talazoparib, which inhibits PARP1 and PARP2 (the latter is linked to toxicity [9]). **e** TMZ treatment of EW-8 cells (0–1 mM) ± *MGMT* or **g** *PARP1* gene knockdown by siRNA (Alamar Blue staining). Student’s paired 2-tailed t-test: *p* = 0.05 (**e**); *p ≤ 0.05 (**g**). **f** MGMT or **h** PARP1 protein downregulation by siRNA (Western blot at 48, 72, 96 h). GAPDH (37 kDa). Beta-actin (43 kDa). NT, no treatment. **i** EW-8 cells ± TMZ treatment (1 mM, 2 h): PARP1 pulldown was followed by PARP1 (top) or MGMT (bottom) immunoblotting. Lanes 1–2: co-immunoprecipitation. Lane 3: IgG1. Lanes 4–5: input. **j** Mean of protein band intensities generated from 3 independent co-immunoprecipitation experiments in (**i**). Student’s paired 2-tailed t-test: *p ≤ 0.05; **p ≤ 0.01 (see reverse co-IP in Additional File 1: Fig. S2c, d). **k** Negative (PARP1-GAPDH) and positive (PARP1-PARP2) interactions by co-immunoprecipitation in EW-8 cells. Samples prepared as in (**i**). IgG control is in middle lane. **l** Representative image of EW-8 cells nuclei staining with Hoechst 33,342 (blue, nuclei), Alexa Fluor 647 (red, MGMT), and Alexa Fluor 488 (green, PARP1). White pixels indicate green and red overlap, i.e., co-localization of PARP1 and MGMT. Top panel, no TMZ. Bottom panel, TMZ at 1 mM for 2 h*.* **m** Scatterplot representing red (MGMT) and green (PARP1) pixel intensities in (**l**); overlap of these colors along the diagonal in the field ‘c’ (~ 45°) corresponds to protein co-localization dots (shown as white pixels). Co-localization analysis was done using CellSens software (v2.1). Images were developed with Fluoview FV3000. **n** Quantification of white-pixel number of co-localized PARP1-MGMT sites in control *vs* TMZ-treated EW-8 cell nuclei. Data from 3 independent experiments were used for the analysis. NT, no treatment. **o** SDS-PAGE and silver staining of protein gel showing PARP1-MGMT interaction by immunoprecipitation assay. Pulldown with full-length PARP1. **p** Purified PARP1 and MGMT protein interaction. Mixed full-length PARP1 and MGMT proteins (1:1) were subjected to co-immunoprecipitation. PARP1 was pulled down with the co-immunoprecipitation specific PARP1 antibody (cst-9532) and immunoblotted with PARP1 (top; cst-9542) or MGMT (bottom; sc-241154) antibodies. IgG control is in the middle lane. **q** Purified N-terminal of PARP1 (aa 1–662) was mixed with full-length MGMT (1:1) and processed for co-immunoprecipitation. Samples prepared as in (**p**). IgG control shown in middle lane. **r** An MST-on time of 10 s analysis of the full-length PARP1 and MGMT protein affinity was performed using Monolith NT.115 at 17% LED power and medium MST power

To test the conjecture of physical interaction between PARP1 and MGMT underlying the linear cellular response, we used co-immunoprecipitation, pulldown, and microscale thermophoresis (MST) analyses. The amount of co-immunoprecipitating proteins became enhanced in the temozolomide-induced EW-8 cells (Fig. 1i–k; Additional File 1: Fig. S2c–e). Consistent with these data, co-localization of these proteins in temozolomide-treated cells was increased by confocal imaging (Fig. 1l–n; Additional File 1: Fig. S2f). Similarly, SDS-PAGE and silver staining of the immunoprecipitates from purified recombinant PARP1 and MGMT proteins revealed the direct interaction between N-terminal PARP1 (aa 1–662) and MGMT proteins (Fig. 1o–q). MST yielded a K_D_ of 165 nM, reflecting a strong purified PARP1 and MGMT affinity (Fig. 1r).

We next asked whether PARP1 can PARylate MGMT, and whether this is one of the interaction mechanisms for these proteins. Total cellular PAR levels were determined by ELISA, and PARylation activity of purified PARP1 was analyzed using synthetic single- and double-strand DNA probes with/without O^6^meG damage, and in the presence/absence of NAD^+^. Importantly, MGMT was PARylated by PARP1, and the strongest increase in MGMT PARylation was observed in the presence of a double-strand DNA-O^6^meG oligo (lanes 9 & 22; consistent with PARP1 auto-modification activation) (Fig. 2a, b; Additional File 1: Fig. S3a, b). In the cellular context, the total PAR signal measured by ELISA was induced by temozolomide treatment (Fig. 2c).

**Fig. 2:**
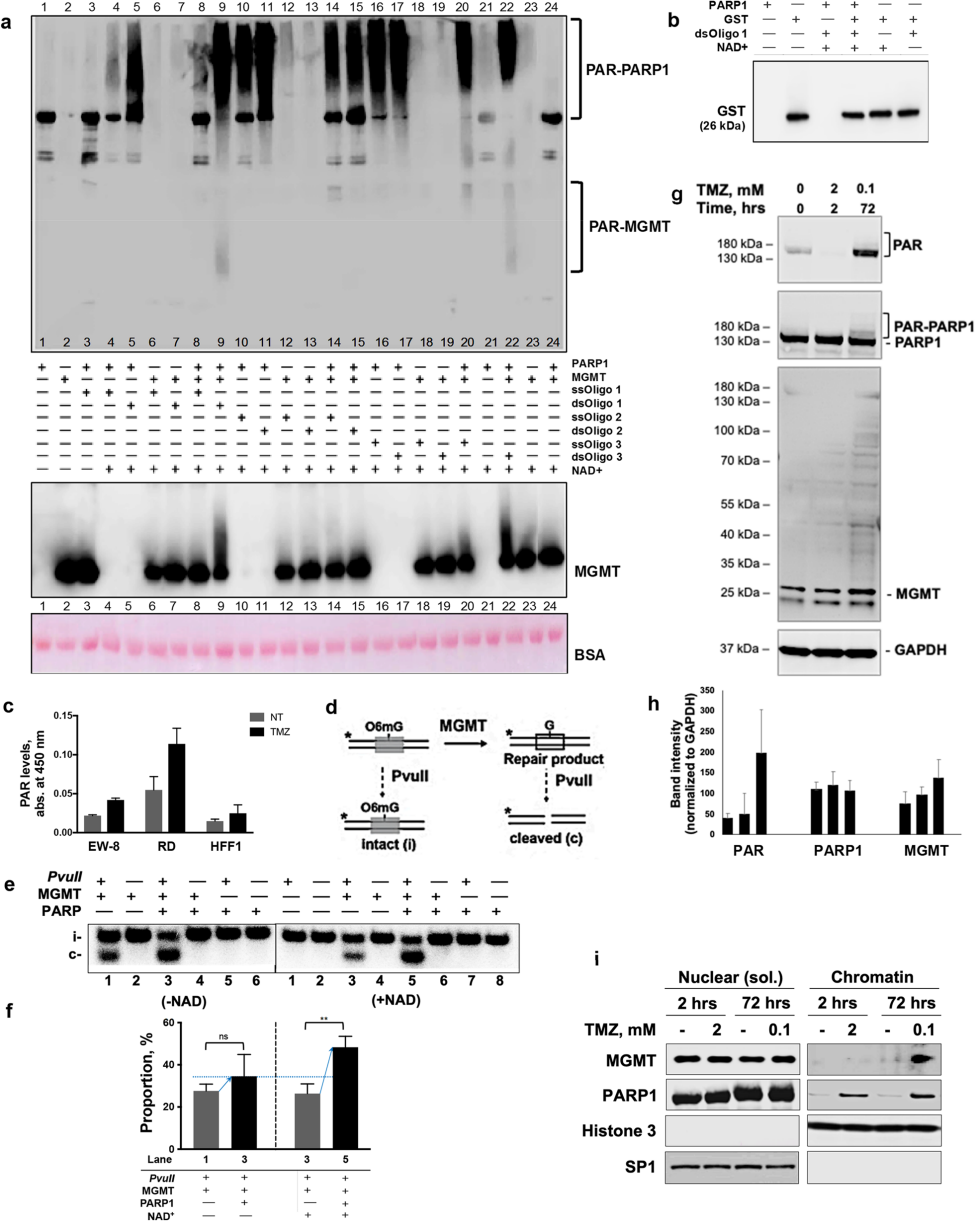
Alkylating DNA damage intensifies O^6^meG repair through PARylation of MGMT. **a** SDS-PAGE/Western blot (top and middle) and BSA Ponceau staining (bottom) for PAR and MGMT. Key: Ss/dsOligo1 is MCAT; ss/dsOligo2 is MGMT-Oligo; ss/dsOligo3 is ss/dsMGMT-O^6^meG. The resulting proteins were detected by SDS-PAGE analysis followed by Western blot for PAR (top) and MGMT (bottom). See PARP1 Western blot in Additional File 1: Fig. S3b. BSA, Ponceau S membrane staining. PAR-PARP1 is auto-PARylated PARP1. PAR-MGMT is PARylated MGMT. **b** Glutathione-S-transferase (GST) is a non-binding substrate of PARP1 and is not PARylated (serves as control). The purified PARP1, GST proteins, NAD^+^, and dsOligo1 were processed as in (**a**). **c** ELISA assay to evaluate PAR levels in Ewing sarcoma EW-8, rhabdomyosarcoma (RD), and fibroblast HFF1 cell lines ± temozolomide treatment (1 mM, 2 h) using SpectraMax M5 plate reader (450 nm). Student’s paired 2-tailed t-test: **p ≤ 0.01. NT, no treatment. TMZ, temozolomide. **d** MGMT repair assay diagram. The repair product is cleavable by *PvuII* restriction digestion. The unrepaired O^6^meG dsDNA (intact, **i.**) and repair product (cleaved, **c.**) can be analyzed by gel electrophoresis. **e** MGMT repair assay. MGMT and PARP1 (6.2 nM) were incubated with MCAT dsDNA for 1 h at 37°C to induce PARylation and then incubated with ^32^P-labeled-O^6^meG-dsDNA (50 nM) for repair reaction. Reaction products were analyzed by *PvuII* treatment followed by PAGE and phosphor-imaging. **f** % of repair results quantified using Image J as a ratio of cleaved band intensity to a sum of intact and cleaved band intensities from (**e**) were plotted (by Prism 8). Stronger increase in DNA cleavage (O^6^meG repair) was observed in the presence of PARP1 and NAD^+^. Student’s paired 2-tailed t-test: **p ≤ 0.01. **g** PARylation activity in EW-8 cells in response to short- (2 mM, 2 h) and long-term (100 μM, 72 h) temozolomide treatment by Western blot for PAR, PARP1, MGMT, and GAPDH proteins. PAR-PARP1 is PARylated PARP1. **h** Quantified band intensities for PAR, PARP1, and MGMT bands normalized to GAPDH levels (*n* = 3) and plotted using Image J. GAPDH (37 kDa) is loading control. **i** Chromatin and nuclear soluble fractions of EW-8 cells treated with temozolomide at 2 mM for 2 h or at 100 μM for 72 h by Western blot. Histone 3 (15 kDa) is chromatin fraction control. SP1 (81 kDa) is nuclear soluble fraction control

To elucidate the significance of MGMT PARylation, the MGMT repair activity was analyzed using *PvuII* restriction digestion in the presence of NAD^+^-dependent ^32^P-labeled-O^6^meG-dsDNA probe and PARP1. MGMT PARylation led to significant NAD^+^-dependent enhancement of O^6^meG repair indicating that PARylation-mediated PARP1-MGMT complex is formed to increase DNA repair (Fig. 2d-f; Additional File 1: Fig. S3c, d). Further, PARylation in EW-8 cells was measured by immunoblotting using short-term (2 mM, 2 h) and more clinically relevant ‘chronic’ (100 μM, 72 h) temozolomide treatment, which induced PARylation and MGMT signals at 100 μM (Fig. 2g–h). Further, temozolomide can stabilize MGMT levels in the global transcription inhibition context (Additional File 1: Fig. S3e) suggesting that de novo MGMT translation does not take place in response to DNA damage. To ascertain whether MGMT PARylation leads to protein stabilization or enhances association with chromatin and/or PARP1, the identification of PARylation sites on MGMT, generation of MGMT mutants that are refractory to PARylation, and extensive analyses of the effect of these mutations on the basal attributes of MGMT is required. Furthermore, the subcellular protein fractionation showed PARP1 and MGMT binding to chromatin under extended temozolomide treatment as reported by others for co-immunoprecipitated glioblastoma cell lysates (Fig. 2i; Additional File 1: Fig. S3f–g) [7]. It is plausible that in glioblastoma cells the sensitization to PARP1 inhibition is linked to BER impairment rather than MGMT activity. In MGMT-deficient gliomas, the DNA mismatch repair can be activated providing an alternative mechanism to O^6^meG repair and cell survival. Consistent with our cell-free data, the fractionation results suggest that temozolomide induces PARP1 and MGMT binding to chromatin, where MGMT responds to clinically relevant ‘chronic’ drug exposure. Finally, we verified that PARP1 and MGMT form a complex in several other cell lines, including rhabdomyosarcoma, rhabdoid tumor, synovial sarcoma, and fibroblasts, indicating that this interaction is not cell-type specific (Additional File 1: Fig. S1e–g).

In summary, we present the first evidence of the direct crosstalk between PARP1 (via BER) and MGMT, which were previously thought to function independently (Additional File 1: Fig. S1h). We showed that PARP1 and MGMT can use either a non-catalytic (DNA-independent) or catalytic (DNA damage-dependent) mechanism of interaction, and the latter increases O^6^meG repair activity through PARP1-mediated MGMT PARylation. Cellular levels of the PARylated MGMT and the MGMT bound to chromatin are enhanced by the clinically relevant ‘chronic’ temozolomide exposure suggesting the PARP1-MGMT-mediated DNA repair takes place during the extended cycles of chemotherapies. Finally, many cancer types and neurodegenerative disorders are dependent on PARP1- and MGMT-mediated repair mechanisms, so our findings provide the rationale to consider the PARP1-MGMT complex as a novel therapeutic target for such diseases.

## Supplementary Information

**Additional file 1.** Supplementary Figures S1, S2, S3, and Methods.

## Data Availability

The datasets used and/or analyzed during the current study are available from the corresponding author on reasonable request.

## Abbreviations

BER: Base excision repair
BRCA: Breast cancer gene
Co-IP: Co-immunoprecipitation
dsDNA: Double-stranded DNA
ELISA: Enzyme-linked immunosorbent assay
In silico: By means of computer simulation
In vitro: Using purified proteins
In vivo: In cellular context
MGMT: O^6^-methylguanine-DNA methyltransferase
MST: Microscale thermophoresis
N^7^meG: N^7^-methylguanine
N^3^meA: N^3^-methyladenine
O^6^meG: O^6^-methylguanine
NAD: Nicotinamide adenine dinucleotide
PAR: Poly(ADP) ribose
PARP1: Poly(ADP) ribose polymerase
PARylation: Poly(ADP) ribosylation
SDS-PAGE: Sodium dodecyl sulfate–polyacrylamide gel electrophoresis
ssDNA: Single-stranded DNA
TLZ: Talazoparib
TMZ: Temozolomide
