# Supplementary material for: PARP1-MGMT complex underpins pathway crosstalk in O6-methylguanine repair
Source: J Hematol Oncol. 2022 Oct 14;15:146. doi: 10.1186/s13045-022-01367-4 (PMC9563463; doi:10.1186/s13045-022-01367-4)
Supplement: Supplementary file 1 — Additional file 1. Supplementary Figures S1, S2, S3, and Methods. [file 13045_2022_1367_MOESM1_ESM.docx]

**Additional FIle 1**

**d**

**f**

**g**

**Olaparib**

**Veliparib**

**b**

**e**

**a**

**c**


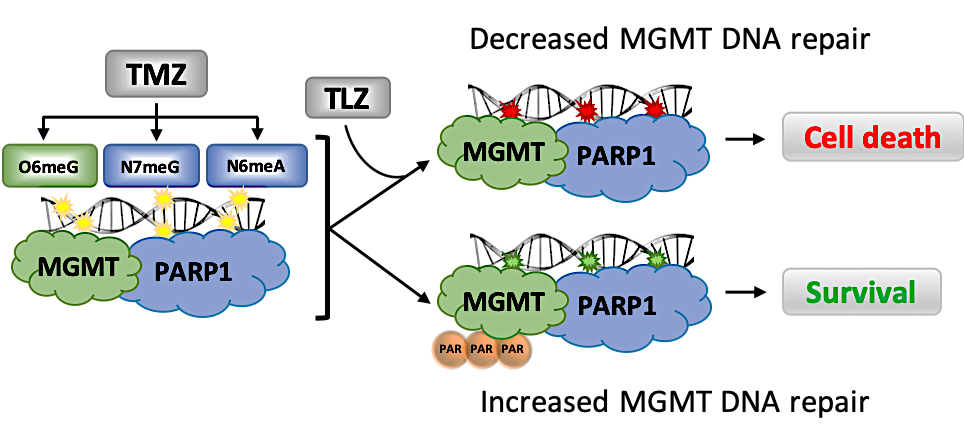


**h**

**d**

**Supplementary Figure S1. Supporting data for Figure 1.** ***(a)*** Survival profiles of EW-8, ES-6, ES-7, and ES-4 Ewing sarcoma cell lines treated with talazoparib (TLZ), temozolomide (TMZ), and O^6^-benzylguanine (O^6^BG) (Alamar Blue staining). P-values are calculated by ANOVA followed with Tukey HST test. ES-4 cells are MGMT deficient and, hence, sensitized to talazoparib and temozolomide. O^6^BG, O^6^-benzylguanine. ***(b)*** Sensitivity to PARP1 inhibitors olaparib (OLP) and veliparib (VLP) in combination with temozolomide and O^6^BG in EW-8 cells. Cells were treated with OLP (1-10 mM) or VLP (1-10 mM) for 96 h; fluorescence signal was measured after incubating cells with Alamar Blue for 2 h. ***(c)*** Normalized RPKM MGMT transcript expression in ES-6, ES-7, EW-8, and ES-4 cells by mRNA sequencing to ES-4 cells have low levels of MGMT transcript due to promoter hypermethylation. ***(d)*** Western blot for basal levels of MGMT and GAPDH (loading control) proteins in 9 Ewing sarcoma cell lines. ES-4 cells do not show detectable MGMT consistent with their mRNA levels in (c). ***(e)*** Rhabdomyosarcoma (RD), rhabdoid tumor (Rh-18), and fibroblast (HFF1) cell lines formed PARP1-MGMT complex upon temozolomide treatment (1 mM, 2 h). PARP1 pulldown was followed by MGMT immunoblotting. IgG1, middle lane. ***(f)*** Synovial sarcoma cells (Aska) form PARP1-MGMT complex. IgG^-^ (no-antibody IgG) validates the IgG signal. Cells were treated as in (e). ***(g)*** MGMT-deficient Rh-28 rhabdomyosarcoma cells do not form PARP1-MGMT complex (co-immunoprecipitation as in (e)). EW-8 cell lysate (far right lane), positive control. ***(h)*** Proposed model of the O^6^meG repair regulation by PARP1. Temozolomide-induced O^6^meG, N^7^meG, and N^3^meA adducts are repaired by PARP1 and MGMT. In the presence of talazoparib, the O^6^meG repair activity is impacted leading to accumulation of DNA damage and cell death. In the absence of talazoparib, MGMT is PARylated by PARP1 leading to increase in O^6^meG repair and cell survival.

**g**

**1 2 3 4 5**

**c**

**e**

**a**

**b**

**d**


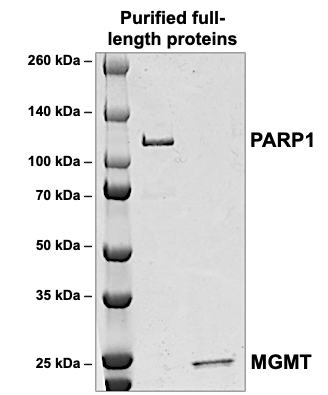


**f**

**Supplementary Figure S2*.* Supporting data for Figure 1. *(a)*** siRNA sequences (n=3) used in Figure 1c-d. Each treatment group was run at n=4. ***(b)*** Standard error of the mean (SEM) calculations for the RNAi data in Figure 1c-d. ***(c)*** Reverse co-immunoprecipitation for MGMT pulldown and PARP1 immunoblotting. EW-8 cells were treated with TMZ (1 mM, 2 h), MGMT was pulled down with MGMT antibody and immunoblotted with PARP1 (top) or MGMT (bottom) antibodies. Lanes 1-2: co-immunoprecipitation. Lane 3: IgG1 control. Lanes 4-5: input. ***(d)*** Mean values of protein band intensities generated from at least 3 representative co-immunoprecipitation experiments with MGMT pulldown and PARP1 immunoblotting*.* ***(e)*** SEM calculations for band intensity values from co-immunoprecipitation experiments (n=3) in EW-8 cell line. Analysis was done using ImageStudioLite-2 software (see Figure 1i-k). ***(f)*** SEM quantification of white-pixel number of co-localized PARP1-MGMT sites in control *vs* temozolomide-treated EW-8 cell nuclei. Data from independent experiments (n=3) were used for quantification of fluorophores’ pixels. ***(g)*** Purified full-length PARP1 (116 kDa) and MGMT (21 kDa) proteins validated by SDS-PAGE.

**g**

**a**

**b**

**c**


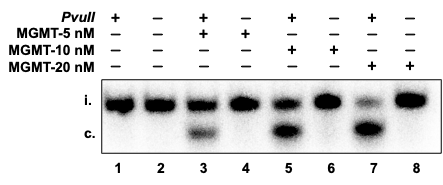

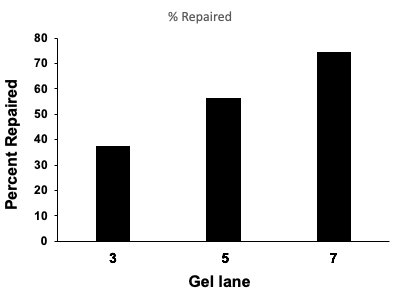


**e**

**d**

**f**

Supplementary Figure S3. Supporting data for Figure 2. *(a)* DNA substrates (0.3 μg/μl) were analyzed by SDS-PAGE followed by staining with SYBR Safe DNA Gel Stain. Lane 1 is ssOligo1; lanes 2-3 are dsOligo1; lane 4 is ssOligo2; lanes 5-6 are dsOligo2; lane 7 is ssOligo3; lanes 8-9 is dsOligo3. *(b)* PARP1 immunoblotting of the reaction mixture of purified PARP1, MGMT proteins, NAD^+^, ssDNA/dsDNA, and BSA (loading control). Ss/dsOligo1 is undamaged MCAT DNA; Ss/dsOligo2 is undamaged MGMT DNA; Ss/dsOligo3 is O^6^meG damaged DNA. The resulting proteins were detected by SDS-PAGE followed by Western blot for PARP1 (see Figure 2a). PARP1 band reduces with the increase in its auto-modification. *(c)* Titration of MGMT protein amount for MGMT repair activity assay (see scheme in Figure 2d): MGMT protein (5, 10, 20 nM) was incubated with ^32^P-labeled-O^6^meG-dsDNA (50 nM) for repair reaction. Reaction products were analyzed by *PvuII* treatment followed by PAGE and phosphor-imaging. *(d)* Quantification measurements for (c) were plotted using Image J. Y axis, % of repair results quantified as ratio of cleaved band intensity to a sum of intact *(i.)* and cleaved *(c.)* bands intensities. *(e)* Western blot of EW-8 cells treated with Actinomycin D (24 h ,158 nM) ± temozolomide (2 h and 4 h, 2 mM). Total cell lysates were probed for MGMT and GAPDH proteins. *(f)* Subcellular cytoplasmic, membrane, and cytoskeletal fractions isolated from EW-8 cells treated with 2 or 0.1 mM of temozolomide for 2 h and 72 h (for Figure 2i). GAPDH (37 kDa), CD99 (32 kDa), and vimentin (57 kDa) loading were evaluated as controls for the cytoplasmic, membrane, and cytoskeletal fractions, respectively. The amount of chromatin-bound MGMT was several folds lower than in other subcellular fractions between independent experiments (n=3) that led to variability in detection levels of the temozolomide-induced MGMT. *(g)* Western blot of chromatin and nuclear soluble subcellular fractions of EW-8 cells treated with temozolomide (72 h, 100 μM), talazoparib (72 h, IC_50_=147.8 nM), and O^6^-benzylguanine (O^6^BG; 72 h, 5 μM). This is an extension for the experiment in Figure 2i supporting our previous results for temozolomide effect and demonstrate the inhibitory effect of O^6^BG on MGMT (also serves as control). Talazoparib did not induce significant changes in either protein or fraction. Histone H3 (15 kDa) is control for chromatin fraction. EWSR1 (85 kDa) is control for nuclear soluble fraction.

**Methods**

**Cell culture**

Ewing sarcoma (ES-4, ES-6, ES-7, EW-8), rhabdomyosarcoma (RD), rhabdoid tumor (Rh-18) (developed in Peter Douglas laboratory at St. Jude), and synovial sarcoma (Aska) cell lines (kindly provided by Yuzuru Shiio) were cultured in RPMI-1640 medium (*SH30027.02,* *HyClone*) supplemented with 10% heat-inactivated FBS (*F-4135,* *Millipore Sigma*). Human fibroblasts (HFF1) (*SCRC-1041, ATCC*) were grown in DMEM medium (*10-013-CV, Corning*) supplemented with 15% heat-inactivated FBS. Cells were maintained at 37°C in a humidified atmosphere with 5% CO_2_. All cell lines are authenticated and mycoplasma-free. They are used in the lab on a continuous basis. Generally, a cell line reaching a passage number over 30 is discontinued and an earlier passage cell line is used instead.

**Cell viability**

The Alamar Blue® assay was used to assess cell viability (*BUF012B*, *BioRad*). Cells were seeded to reach 20-40% confluency (lower confluency for rapidly growing cells). Talazoparib (*HY-16106, MedChemExpress),* temozolomide (*HY-17364/CS-0943, MedChemExpress*), and O^6^-benzylguanine (*B2292, Sigma Aldrich*) were added to wells 24 h after cell seeding, and incubated for 96 h. After 2 h incubation of cells in 24-well plates (1 ml of culture medium per well), 10% v/v Alamar Blue (100 μl) was added and fluorescence was measured (excitation 530 nm, emission 590 nm). Wells containing RPMI-1640 (*SH30027.02, Hyclone*), 10% FBS (*Millipore Sigma*) and untreated cells, and 10% v/v Alamar blue were used as positive controls. Wells with culture medium without cells containing 10% v/v Alamar Blue were negative controls. Fluorescence was recorded on a Spectra Max plate reader, using the Alamar Blue protocol provided by *Softmax Software*. Statistical analyses and curve plotting (4-parameter polynomial analysis) were performed using standard equations included in the GraphPad Prism 7.0c package (*GraphPad Software Inc., USA*).

**RNAi high-throughput screen**

ES-7 and EW-8 Ewing sarcoma cell lines were plated in a 384-well plate and siRNA against *PARP1* and *MGMT* genes (3 siRNAs per gene; *Thermo,* Silencer Select Whole Genome siRNA library) were added to the wells for 48 h. No siRNA control and no treatment (temozolomide) control groups were included in the analysis. Next, cells were treated with temozolomide (IC_50_) for additional 72 h, after which cell viability was quantitatively measured using ATP-lite assay (*Perkin Elmer,* 6016739). Cells for each treatment group were plated in quadruplicates for statistical power. Mean IC_50_ values were calculated for 3 siRNAs per gene run in triplicate. Selected gene knockdowns were performed using the same siRNAs against *MGMT* (s8750, s8752, s224042) and *PARP1* (s1099, s1097, s1098) genes. The siRNA sequences are listed in the **Figure S2a**.

**mRNA sequencing**

At least 500 mg of total RNA was isolated from each of the Ewing sarcoma cell lines (ES-4, ES-6, ES-7, EW-8) using Qiagen RNeasy Mini Kit (*74104, Qiagen*) and Trizol (*15596026, Thermo*) and used for RNAseq library preparation by following the Illumina TruSeq stranded mRNA sample preparation guide (*Illumina, CA*). The QC was done by nanodrop UV measurement, and by running a standard 1% agarose gel and bioanalyzer. The poly-A containing mRNA molecules were purified using poly-T oligo-attached magnetic beads, then fragmented RNA into small pieces, copied into first strand cDNA using reverse transcriptase and random primers, followed by the 2^nd^ strand cDNA synthesis using DNA Polymerase I and RNase H. After the end repair process, ligation of the adapters, the PCR was performed to create the final RNAseq library, which was then pooled for cBot amplification and subsequently sequenced with the Illumina HiSeq 2000 platform using 100 bp paired-end module at Greehey Children’s Cancer Research Institute’s Genome Sequencing Facility. Upon obtaining the sequence reads, the samples were aligned to USCS hg19 human genome built using TopHat2 aligner, and expression levels (both in read counts and in Reads Per Kilobase of transcript (RPKM)) were summarized using HTSeq with all RefSeq genes.

**Gene knockdown by siRNA**

Gene-specific siRNAs (mix of 4 sequences) for PARP1 (*Horizon*, *L-00656-03-0005*) and MGMT (*Horizon*, *L-008856-01-0005*) were transfected into EW-8 cells using Lipofectmine RNAiMAX Reagent (*Thermo, 13778*). Manufacturer’s protocol for reverse transfection was followed. Transfections were conducted in 6-well plates, and siRNA concentration of 10 pmol/well used. Cells were harvested at 48 h, 72 h, and 96 h post-transfection and lysed for Western blot to confirm the knockdowns.

**Protein expression and purification**

Expression and purification of PARP1 and MGMT were performed following the protocol described previously with minor modifications (10, 11). Plasmids for full-length PARP1 and N-PARP1 (1-662 aa) protein constructs were kindly provided by John Pascal*.* For the full-length His^6^-PARP1, the protein was overexpressed using *Escherichia coli* strain BLR (DE3) pRARE *(69053-3, Novagen)*. Transformed cells were grown in 2 x LB medium and protein was induced with 0.2 mM isopropyl ß-D-thiogalactopyranoside and 0.1 mM of ZnCl_2_ at 16^o^C for 20 h. Cell lysate was prepared by sonication and clarified by ultracentrifugation. His^6^-PARP1 protein was purified with 2 ml Ni-NTA agarose *(30230, Qiagen)*, 1 ml HiTrap Heparin HP *(17040701, GE Healthcare)*, and HiTrap SP HP *(17115201*, *GE Healthcare)*.

**Microscale thermophoresis**

Full-length PARP1 protein (*4668-02K-01, Trevigen*) primary amines were labeled using the Protein Labeling Kit RED-NHS 2^nd^ Generation (*MO-L011, NanoTemper Technologies*). The labeling reaction was performed according to the manufacturer’s instructions using the labeling buffer provided in the kit, applying a concentration of 2.7 μM protein (molar dye: protein ratio ≈ 5:1) at room temperature for 30 min in the dark. Unreacted dye was removed with the dye removal column included in the kit and equilibrated with PBS (pH 7.5). The labeled protein PARP1 was adjusted to 100 nM with PBS supplemented with 0.005% of Tween-20. The ligand MGMT (*NBC1-18534, Novus Biologicals*) was dissolved in PBS supplemented with 0.005% of Tween-20, and a series of twelve 1:1 dilutions were prepared using the same buffer, producing ligand concentrations ranging from 13 nM to 21 μM. For measurement, each ligand dilution was mixed with one volume of labeled PARP1, yielding a final concentration of PARP1 of 50 nM and final ligand concentrations of 6.5 nM to 10.5 μM. After 20 min, the samples were loaded into Monolith NT.Automated Premium Capillary Chips (*MO-AK005, NanoTemper Technologies*). Microscale thermophoresis was measured using a Monolith NT.Automated instrument (*NanoTemper Technologies*) at an ambient temperature of 25°C. Instrument parameters were adjusted to 17% excitation and medium power. Data from 3 independently pipetted measurements were analyzed (*MO.Affinity Analysis software version 2.3, NanoTemper Technologies*) using the signal from an instrument-on time of 10 s.

**MGMT repair activity assay with purified proteins**

The unrepaired O^6^meG dsDNA (intact) and its repair product (cleaved) were analyzed by gel electrophoresis. The master mix included 5x reaction buffer (10 μl of 175 mM Tris-HCl, pH 7.5, 5 mM DTT), H_2_O (14.5 μl), MgCl_2_ (5 μl of 10 mM), KCl (3 μl of 1 M), BSA (2.5 μl of 2 mg/ml), NAD+ (5 μl of 50 mM), MGMT, and PARP1 proteins (5 μl of 500 nM) that were incubated with MCAT dsDNA (5 μl of 3.6 μM) for 1 h at 37°C to induce PARylation and then incubated with ^32^P-O^6^meG-dsDNA (50 nM) for MGMT mediated repair reaction. Reaction products were analyzed by *PvuII* treatment (0.5 units/μl) in buffer G (*ThermoFisher*) followed by PAGE and phosphor-imaging. Percent of repair was quantified as ratio of cleaved band intensity to a sum of intact and cleaved band intensities and were plotted using Image J software.

**PARylation assay with purified proteins**

PARylation assay was performed according to the protocol adapted from (12) with minor modification. The PARylation reactions were performed at room temperature in 20 μl of 20 mM Tris-HCl, pH 7.5, 50 mM KCl, 7.5 mM MgCl_2_, and 1 mM DTT. Full-length PARP1 (0.62 μM), MGMT, or GST (GST was generously provided by Yuzuru Shiio) purified proteins were first preincubated with 1 μM of a synthetic single-strand oligomer Oligo1 (MCAT): 5′-GAGTGTTGCATTCCTCTCTGGGCGCCGGGC*Aggta*CCTGCTG-3’ (*Integrated DNA Technologies*), Oligo2 (MGMT): 5′-GCCCGGCCAGCTGCAGTT-3′, Oligo3 (O^6^meG-MGMT): 5′-GCCCGGCCAGCTXCAGTT-3′ (X=O^6^meG), or corresponding double-strand oligomers, for 20 min. BSA protein (0.6 μM) was included in the mixture as loading control, and levels of BSA protein were captured by the Ponceau S staining of the membrane. NAD^+^ (5 mM) was then added to the reaction, and the mixture was incubated for 1 h. The reaction was stopped by the addition of SDS-loading buffer containing 0.1 M EDTA. The samples were separated using 4-12% NuPAGE SDS gel (*NP0321, Invitrogen*), and immunoblotted. Transferred membrane was then incubated with primary antibodies against PAR, PARP1, and MGMT. Double-strand DNA (dsDNA) were made by annealing of an equal molar of 2 complementary oligomers in 1x NEB3 restriction endonuclease buffer *(B7203, New England BioLabs*) using a temperature gradient from 94^o^C to 30^o^C (-0.5^o^C/min). Formation of dsDNA was confirmed using PAGE and staining with SYBR Safe DNA Gel Stain (*S33102, Invitrogen*).

**Protein extraction and immunoblotting**

Cells were lysed and total proteins were extracted using RIPA buffer (*89900, Pierce*) with 10% Halt protease, phosphatase inhibitor cocktail (*1861281, Thermo Fisher Scientific*), and 10% PMSF (*P7626, Sigma Aldrich*) according to standard protocols. Actinomycin D was added prior to cell lysis (158 nM for 24 h) (*A9415, Sigma-Aldrich*). In brief, cells were incubated on ice with lysing buffer for 5 min, then collected into Eppendorf tubes, and centrifuged at 14,000 rpm for 10 min. Samples were separated on a 4-12% gradient gel (*NP0321, Invitrogen*) and transferred onto a PVDF or nitrocellulose membrane. Membranes were blocked with 3% BSA in TBS-T for 1 h at room temperature, then incubated with primary antibody overnight at 4^o^C on a cold room shaker. After secondary antibody incubation and washing, membranes were developed using enhanced chemiluminescence (*NEL103001EA*, *PerkinElmer*).

**Co-immunoprecipitation**

Cells were grown to near confluency in 10 cm dishes. Whole cell lysates were prepared using IP lysis buffer (*87787*, *Pierce*) supplemented with Halt protease and phosphatase inhibitor (*1861281*, *Thermo Fisher Scientific*) plus PMSF (*P-7626*, *Millipore Sigma*), according to standard protocols. Co-immunoprecipitation was done with endogenous and purified PARP1 and MGMT proteins. For purified proteins, the constructs were mixed 1:1 (2 µg total protein concentration each). Pulldowns were performed using SureBeads protein G magnetic beads (*161-4023, BioRad*) according to the manufacturer’s protocol. Bound complexes were eluted with Invitrogen NuPage loading buffer (*NP0007, Invitrogen*) and then evaluated by Western blotting.

**Immunostaining and confocal imaging**

EW-8 cells (1x10^5^) were plated into 24-well plates with glass coverslips in sterile conditions and incubated for 48 h at 37^o^C for cells to settle. Cells were treated with 1 mM of temozolomide for 2 h, and then fixed with 4% formaldehyde for 15 min at room temperature. After blocking for 1 h with blocking buffer (1 x PBS/5% normal goat serum/0.3% Triton x-100) cells were incubated with primary PARP1 and MGMT antibodies overnight at 4^o^C on a cold room shaker. The following day, cell nuclei were stained with Hoechst 33342 (blue) and incubated with fluorochrome-conjugated secondary antibodies against PARP1 (green) and MGMT (red) for 2 h at room temperature. Images were captured with an Olympus I x 80 microscope at 100 X magnification. Images (Z-stacks) were developed and analyzed with Fluoview and CellSens software (v2.1) to identify pixels with a high degree of co-localization (high intensity of staining for both fluorophores) using the built-in co-localization algorithm.

**Antibodies**

The following antibodies were used for Western blotting, immunoprecipitation, immunostaining, and PAR activity assays: anti-MGMT (*sc-271154, Santa Cruz Biotechnology; cst-2739, Cell Signaling Technology*), anti-PARP1 (*cst-9532, cst-9542, Cell Signaling Technology),* anti-PAR (*4335-MC-100, Trevigen/4335-MC-100, R&D*)*,* anti-IgG (*cst-2729s, Cell Signaling Technology*), anti-IgG1 (*cst-5415, Cell Signaling Technology*), anti-GAPDH (*cst-5174S, Cell Signaling Technology*), anti-beta-Actin (*sc-47778, Santa Cruz Biotechnology*), anti-PARP2 (*sc-393343, Santa Cruz Biotechnology*), anti-GST (*A190-122A, Bethyl Laboratories*), anti-Histone 3 (*4499S, Cell Signaling),* anti-SP1 (*9389S, Cell Signaling*), anti-EWSR1 (*cst-11910, Cell Signaling Technology*). Chemiluminescent secondary antibodies: anti-rabbit *(cst-7074S,* *Cell Signaling Technology)* and anti-mouse *(cst-7076S,* *Cell Signaling Technology)* IgG HRP-linked antibodies*.* Fluorochrome-conjugated secondary antibodies: anti-PARP1 Alexa Fluor 488 (*ab150077, Abcam*)*,* anti-MGMT Alexa Fluor 647 (*ab150115,* *Abcam*).

**ELISA for PAR levels**

The PAR was quantitatively measured per manufacturer instructions of the ELISA kit (*XDN-5114, Cell Biolabs, Inc.).* Briefly, cells were washed once with PBS containing 1X PARP inhibitor (1 mg/mL 3-AB), and 1 ml of RIPA buffer containing 1x PARP inhibitor (1 mg/mL 3-AB) was added to every 10 cm culture dish. After incubation for 10-20 min, cells were detached, centrifuged at 10,000 g for 10 min at 4°C. The supernatant with added SDS (final concentration of 1%) was boiled (100°C) for 5 min, cooled on ice after boiling and then centrifuged at 10,000 g for 5 min. These supernatants (100 μl) or PAR standards were added to a monoclonal anti-PAR antibody pre-coated microplate. The series of incubations were done per manufacturer’s instructions. Final reaction was measured with a colorimetric plate reader at 450 nm wavelength. The PAR content in unknown samples was determined by comparing the absorbance with that of the PAR polymer standard curve.

**Subcellular Protein Fractionation**

Isolation of chromatin and nuclear soluble fractions was performed to enrich for cytoplasmic, membrane-bound, soluble nuclear, chromatin-bound, and cytoskeletal proteins using the Subcellular Protein Fractionation Kit for Cultured Cells (*78840, Thermo Fisher Scientific*) according to the manufacturer’s protocol. Briefly, cells were plated in 6-well plates, allowed to adhere overnight and then treated with temozolomide (IC_10_ for 72 h or IC_90_ for 2 h), talazoparib (IC_10_ for 72 h or IC_90_ for 2 h), O^6^-benzylguanine (5 µM for 2 h), or vehicle (DMSO) prior to subcellular fractionation. Fractionation efficiency was evaluated by immunoblotting for markers of cytoplasmic (*GAPDH; 2118L, Cell Signaling Technology*), membrane-bound (CD99; *PA5-32337, Invitrogen*), soluble nuclear (SP1 and EWSR1, *9389S and 11910, Cell Signaling Technology*), chromatin-bound (Histone 3; *4499S, Cell Signaling Technology*) and cytoskeletal (vimentin; *MA5-11883, Invitrogen*) proteins.

**Statistical analysis**

Unless otherwise noted, statistical significance (p-value) in this work was determined by paired/not-paired, one/two-tailed Student’s t-test, and for multiple factors (such as dose-responses with multiple compound treatments) we use *n*-way ANOVA test followed by Tukey’s Honest Significant Difference (HSD) method (R, http://www.R-project.org). Error bars represent the standard error of the mean (SEM).
